# Matrix stiffness mechanosensing modulates the expression and distribution of transcription factors in Schwann cells

**DOI:** 10.1002/btm2.10257

**Published:** 2021-09-21

**Authors:** Gonzalo Rosso, Daniel Wehner, Christine Schweitzer, Stephanie Möllmert, Elisabeth Sock, Jochen Guck, Victor Shahin

**Affiliations:** ^1^ Max Planck Institute for the Science of Light Erlangen Germany; ^2^ Max‐Planck‐Zentrum für Physik und Medizin Erlangen Germany; ^3^ Institute of Physiology II, University of Münster Münster Germany; ^4^ Institute of Biochemistry, FAU Erlangen‐Nürnberg Erlangen Germany; ^5^ Department of Physics FAU Erlangen‐Nürnberg Erlangen Germany

**Keywords:** cell plasticity, extracellular matrix stiffness, mechanosensing, nerve regeneration, neuron, Schwann cell

## Abstract

After peripheral nerve injury, mature Schwann cells (SCs) de‐differentiate and undergo cell reprogramming to convert into a specialized cell repair phenotype that promotes nerve regeneration. Reprogramming of SCs into the repair phenotype is tightly controlled at the genome level and includes downregulation of pro‐myelinating genes and activation of nerve repair‐associated genes. Nerve injuries induce not only biochemical but also mechanical changes in the tissue architecture which impact SCs. Recently, we showed that SCs mechanically sense the stiffness of the extracellular matrix and that SC mechanosensitivity modulates their morphology and migratory behavior. Here, we explore the expression levels of key transcription factors and myelin‐associated genes in SCs, and the outgrowth of primary dorsal root ganglion (DRG) neurites, in response to changes in the stiffness of generated matrices. The selected stiffness range matches the physiological conditions of both utilized cell types as determined in our previous investigations. We find that stiffer matrices induce upregulation of the expression of transcription factors Sox2, Oct6, and Krox20, and concomitantly reduce the expression of the repair‐associated transcription factor c‐Jun, suggesting a link between SC substrate mechanosensing and gene expression regulation. Likewise, DRG neurite outgrowth correlates with substrate stiffness. The remarkable intrinsic physiological plasticity of SCs, and the mechanosensitivity of SCs and neurites, may be exploited in the design of bioengineered scaffolds that promote nerve regeneration upon injury.

## 1 INTRODUCTION

Schwann cells (SCs) are of fundamental physiological importance for the peripheral nervous system (PNS). Their remarkable biological plasticity enables them to rapidly adapt to tissues beyond the PNS microenvironment, as they have the capacity to form myelin around axons after transplantation into the central nervous system (CNS) spinal cord.^1^ These striking properties make SCs a powerful candidate for cell‐based PNS and CNS regenerative therapies.^2^ SC plasticity is reflected by the fact that they undergo significant morphological transformations during PNS development and repair among others.^3^, ^4^ Apart from their canonical role of myelin formation around peripheral axons, SCs clear debris from lesion sites after nerve damage and promote nerve regeneration via diverse interactions with their microenvironment.^5^, ^6^ It is well documented that nerve regeneration is associated with changes in the biochemical properties of the involved structures including SCs. Compelling evidence shows that the nerve lesion site also undergoes significant changes in the biomechanical properties of acellular and cellular constituents that are crucial for nerve regeneration, but the underlying mechanisms remain unclear.

Upon nerve injury, mature SCs dedifferentiate and reprogram into a repair SC phenotype. Repair SCs are characterized by downregulation of the expression of pro‐myelinating genes, such as Krox20, and the upregulation of the transcription factor c‐Jun, a key regenerative marker that promotes nerve regeneration.^7^ Later on, SCs transform into a pro‐myelinating phenotype to re‐myelinate regenerated axons. During the differentiation and dedifferentiation phases, the expression profile of SC‐specific markers changes.^5^, ^8^ We have recently shown that mechanosensing of the extracellular matrix (ECM) impacts physiological processes including embryonic outgrowth of neurites from dorsal root ganglions (DRGs), as well as the spreading area, migration velocity, and biomechanical properties of SCs.^9^ It remained unexplored, however, whether ECM stiffness mechanosensing affects SC differentiation and the regulation of specific phenotypes. In the present work, we generated elastic substrates that cover the physiological range of nerve tissue stiffness to investigate the modulation of SC stage‐specific markers, including c‐Jun, Krox20, Oct6, and Sox2. Here, we show that soft matrices upregulate the expression of the repair‐associated transcription factor c‐Jun, whereas stiff substrates upregulate the expression of transcription factors Sox2, Oct6, and Krox20, suggesting a link between substrate mechanosensing and regulation of gene expression in SCs. Beyond refining our neurophysiological knowledge of the PNS, our findings may be exploited to advance clinical nerve regeneration. They may also be of some relevance for the priming of SC phenotypes/functions for possible future application in the PNS and potentially in the CNS.

## 2 RESULTS AND DISCUSSION

### 2.1 YAP nucleo‐cytoplasmic shuttling in SCs is regulated by ECM stiffness

**FIGURE 1:**
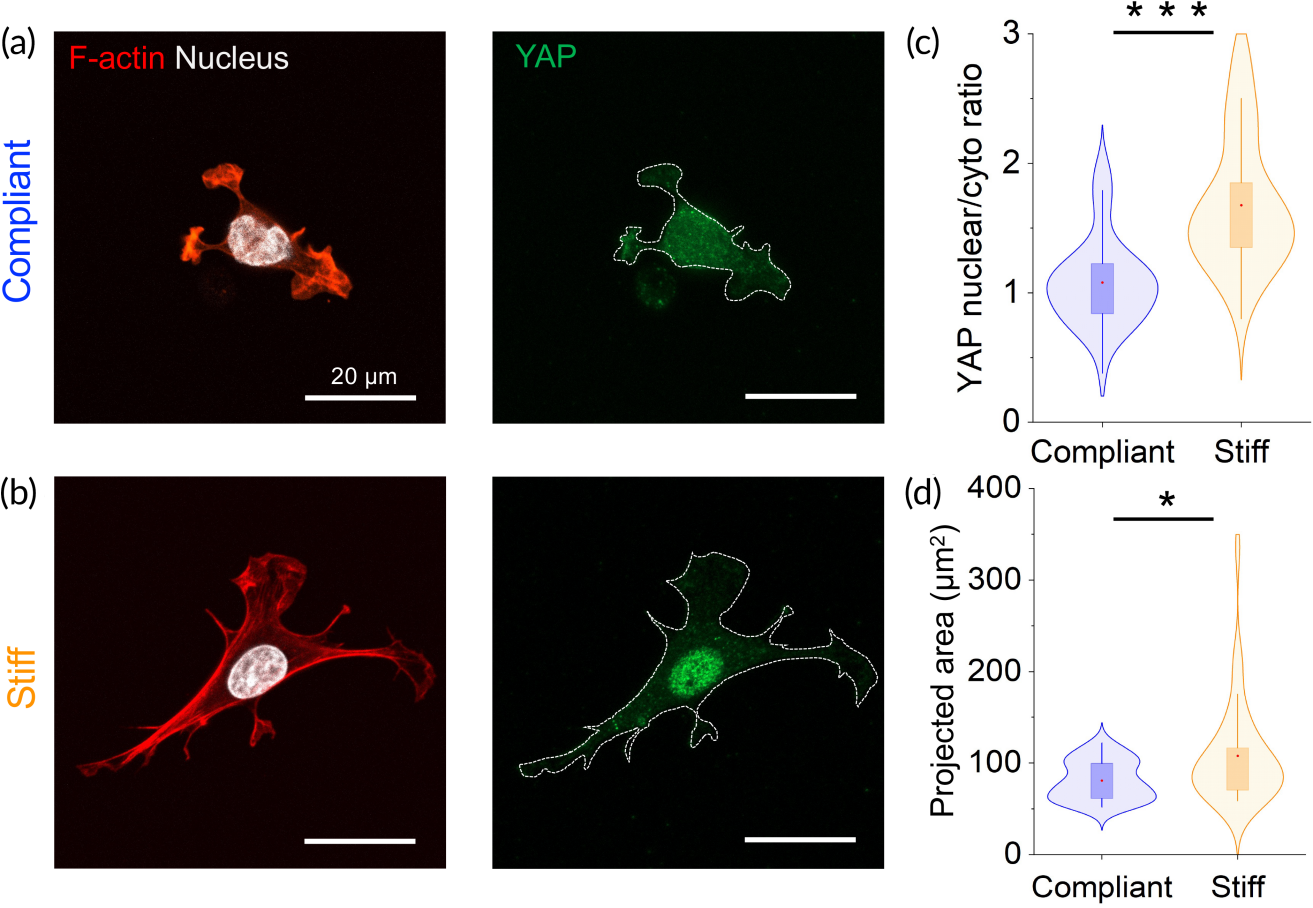
Nucleo‐cytoplasmic YAP localization in SCs is modulated by substrate stiffness. Labeling of YAP (green) on SCs seeded on a compliant (a) and stiff (b) laminin‐coated PAAm substrate. (c) Quantification of nuclear/cytoplasmic ratio. (d) Projected SC area. Cells were stained with DAPI (white) and rhodamine‐phalloidin (red) for nucleus and F‐actin labels, respectively. *P <.05, Mann–Whitney test. n = 50 cells for each substrate. Abbreviation: SC, Schwann cell

Our recent study demonstrates that SCs are highly mechanosensitive, and that ECM stiffness directs different physiological responses in SCs, which include morphological changes, cell‐ECM adhesion, motility, and cell mechanics.^9^ It remains an open question, however, whether the mechanosensitivity of SCs also modulates their biochemistry and differentiation stages. To address this question, we generated ECM‐coated polyacrylamide (PAAm) substrates with constant biochemical composition but varied biomechanical properties (Young's modulus) (see Figure S1, supporting information). Substrate stiffness was appropriately selected to cover the physiological stiffness range of the native microenvironment of SCs and axons.^10^, ^11^ We coated the generated elastic substrates with the ECM protein laminin owing to its importance in the peripheral nerve microenvironment, and its essential role in mechanosensing and mechanotransduction.^12^ Mechanosensing of the ECM stiffness is a key player in mechanotransduction pathways, including signaling via the serum response factor, NF‐kB, zyxin/paxillin, integrin, E‐cadherin, Wnt, and the Hippo‐signaling pathway among others.^13^, ^14^, ^15^ In the present work, we analyzed in nerve‐derived isolated SCs the modulation of the transcriptional regulator Yap, a mechano‐transducer of the Hippo pathway, in response to changes in ECM stiffness. Figure 1 shows representative confocal images of SCs, seeded on laminin‐coated substrates. YAP (green), nuclei (white), and cytoskeletal F‐actin are visualized following staining with anti‐Yap antibody, DAPI, and rhodamine‐phalloidin, respectively. We found that in SCs exposed to compliant environments (n = 50) of 1.1 kPa, YAP is localized mainly in the cytoplasm, whereas it shuttles to the nucleus when cells are exposed to stiffer (27.7 kPa) substrates (n = 50) [Figure 1(a–c)].

The accumulation of YAP in the nucleus is accompanied by a significant increase in SC spreading area [Figure 1(d)]. These results support previous data showing that SCs sense and respond to changes in matrix rigidity by modulating the nucleocytoplasmic transport of YAP/TAZ.^16^ In previous studies, we measured the stiffness changes in the nerve microenvironment during PNS development and disease, using atomic force microscopy.^30,31^ We demonstrated that the basal lamina, a special type of ECM, conveys crucial biomechanical resilience to myelinated axons,^10^ and that it is a major contributor to the overall stiffness of the nerve tissue microenvironment during development and maturation.^11^ SCs interact not only physiologically but also biomechanically with the basal lamina. This interaction significantly impacts the behavior of SCs, which includes their interaction with axons, and eventually myelination.^17^, ^18^ Hence, ECM stiffness and the mechanosensitivity of SCs are of particular importance for the physiological functions of SCs. Next, we set out to investigate the influence of matrix stiffness on the regulation of protein expression profiles in SCs.

### 2.2 Matrix stiffness modulates the expression of pro‐myelin transcription factors

**FIGURE 2:**
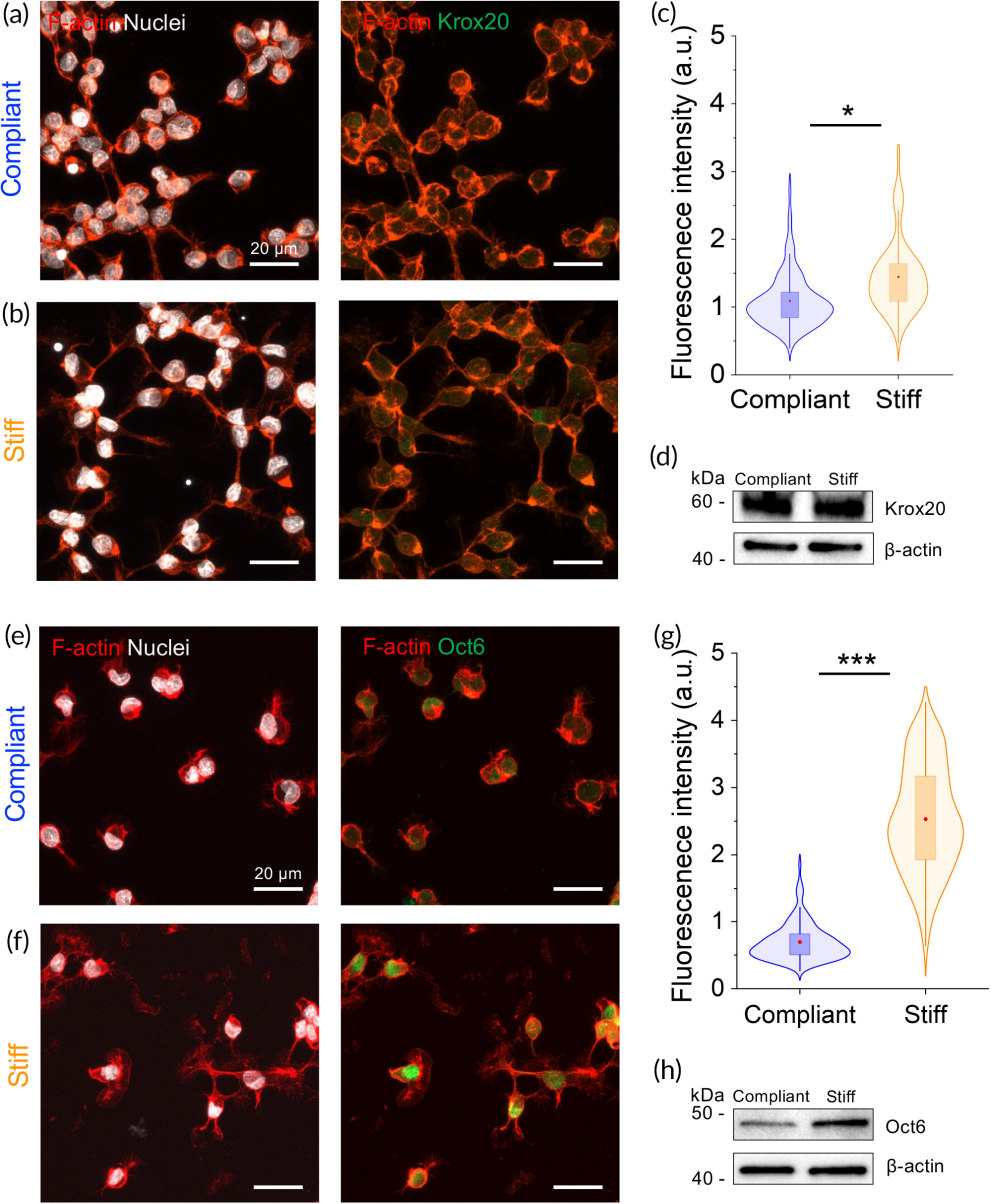
Expression and localization of pro‐myelinating transcription factors in SCs is modulated by substrate stiffness. Representative confocal images showing labeling of Krox20 and Oct6 on SCs seeded on compliant (a and e) and stiff (b and f) substrates. Quantification of fluorescence signal of nuclear Krox20 (c) and Oct6 (g). Western blot showing expression of levels of Krox20 (d) and Oct6 (h). Actin cytoskeleton and nucleus labeled in red and white, respectively. *P < .05 and ***P < .0001, Mann–Whitney test. Krox20: n = 349 and n = 235 cells for compliant and stiff substrates, respectively. Oct6: n = 156 and n = 183 cells for compliant and stiff substrates, respectively. Abbreviation: SC, Schwann cell

Recent in vivo studies have shown that transcriptional regulators YAP/TAZ are involved in the upstream regulation of myelin genes in mouse peripheral nerves.^19^ The activation of YAP/TAZ signaling pathway in SCs is regulated via integrin‐mediated signaling and G‐protein mechanism. Once activated, YAP and TAZ bind to DNA binding partners such as TEAD1‐4, which modulate the proliferation and expression of myelin genes along with ERG2 and Sox10.^16^, ^20^ It remains unclear, however, whether physical cues affect SCs differentiation. How physical cues modulate different SC phenotypes has important implications for nerve regeneration strategies, based on bioengineered nerve scaffolds containing cellular components, which mostly include SCs. We studied in SCs the expression levels of the transcription factor Krox20 (also known as Egr2), a master regulator of the onset of myelination in the PNS.^21^ Krox20 protein levels were examined by immunocytochemistry in nuclei of SCs, which were kept for 48 hours on substrates possessing PNS‐matched stiffness values. Figure 2 shows fluorescent microscopy images of immunostained SCs on compliant [Figure 2(a)] and stiff [Figure 2(b)] substrates. Fluorescent intensity analysis indicates a modest but significant increase in the nuclear expression of Krox20 in SCs cultured on stiff substrates (n = 236 cells), as compared to compliant substrates (n = 349 cells) [Figure 2(c)]. Western blot analysis of total protein from cell lysates showed no apparent difference in Krox20 expression when comparing SCs on compliant and stiff substrates [Figure 2(d)].

The transcription factor Oct6 (also known as SCIP/Tst1) is an upstream regulator of Krox20 in SCs and is considered necessary for the transition from nonmyelinating to the myelinating stage in peripheral nerves.^22^ We studied the expression levels of Oct6 transcription factor in SCs seeded on compliant [Figure 2(e)] and stiff [Figure 2(f)] laminin‐coated substrates within the PNS stiffness range. Cells were counterstained with DAPI (white) and rhodamine‐phalloidin (red) for nuclei and cytoskeletal F‐actin visualization, respectively. The immunocytochemistry analysis of Oct6 expression in Figure 2(g) shows that the latter has a similar tendency to the transcription regulatory factor Krox20. We found that stiffer substrates significantly promote the upregulation of the transcription factor Oct6 in SCs nuclei (almost 3.6‐fold, n = 183 cells), compared to nuclei of SCs exposed to compliant substrates (n = 156 cells) [Figure 2(g)]. Higher levels of Oct6 in SCs on stiffer matrices were also confirmed by Western blot [Figure 2(h)]. Taken together, these results indicate that the expression of pro‐myelin genes such as Krox20 and its upstream regulator Oct6 are sensitive to mechanical signals, and are upregulated in stiffer microenvironments.

### 2.3 Effect of substrate stiffness on SC plasticity and regeneration

**FIGURE 3:**
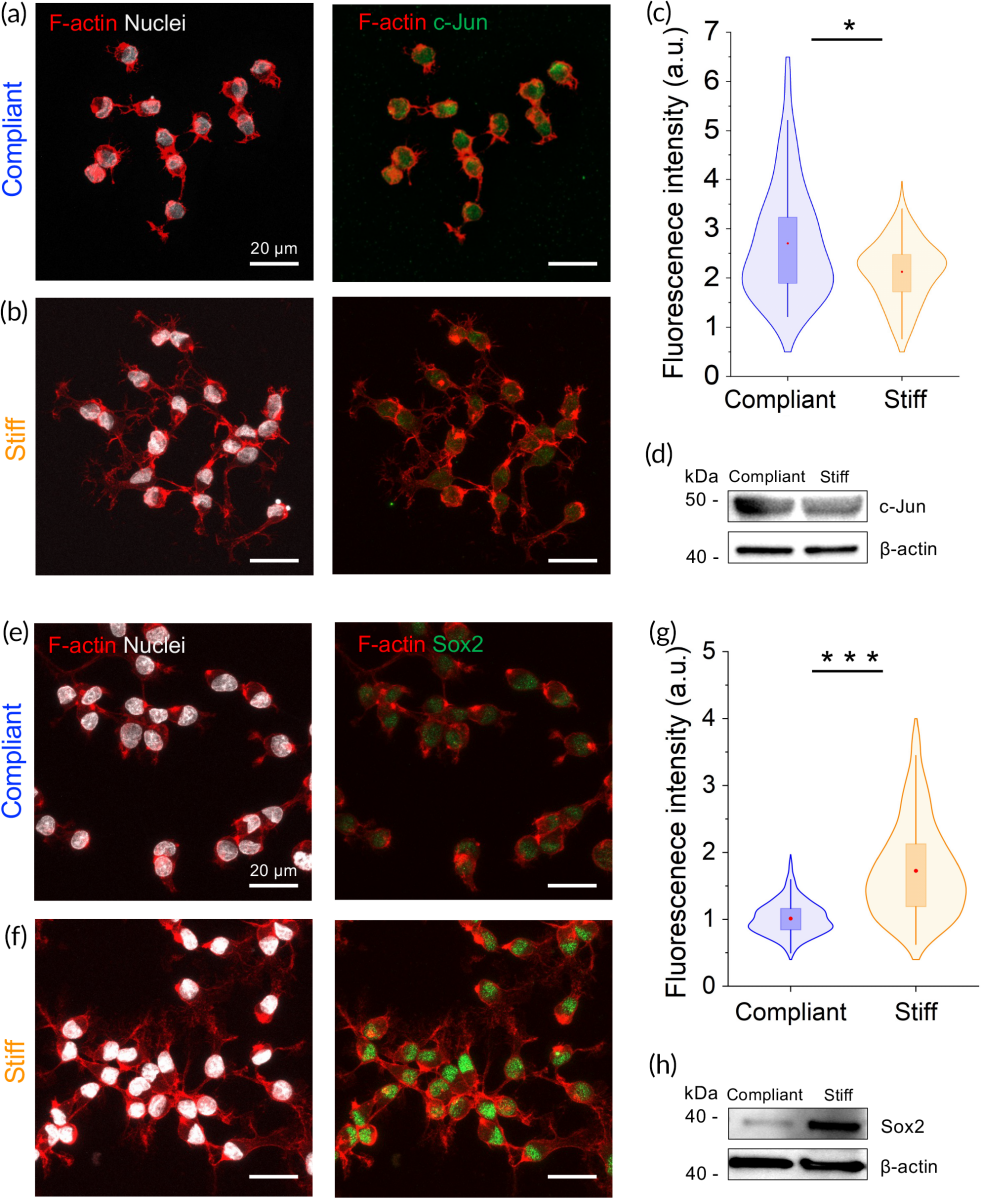
Expression of pro‐regenerating/repair transcription factor in SCs is modulated by substrate stiffness. Representative confocal images showing labeling of c‐Jun and Sox2 on SCs seeded on a compliant (a and e) and stiff (b and f) substrates. Fluorescent quantification of nuclear c‐Jun (c) and Sox2 (g). Western blot showing expression of levels of c‐Jun (d) and Sox2 (h). Actin cytoskeleton and nucleus labeled in red and white, respectively. *P < .05 and ***P < .0001, Mann–Whitney test. c‐Jun: n = 125 and n = 183 cells for compliant and stiff substrates, respectively. Sox2: n = 200 and n = 235 cells for compliant and stiff substrates, respectively. Abbreviation: SC, Schwann cell

As previously mentioned, during nerve development the biochemical and mechanical properties of the nerve tissue architecture around SCs significantly transform, mainly by the deposition of basal lamina and the communication with maturing axons.^3^, ^18^ SCs possess the remarkable capacity to adapt to dynamic changes in their microenvironment, and the ability to switch between different differentiation states during PNS development. This striking cellular plasticity becomes even more remarkable upon nerve injury, where highly differentiated SCs lose their elongated morphology and downregulate the expression of myelin‐associated genes, such as *Krox20*, Myelin basic protein (*Mbp*), Myelin protein zero (*Mpz*), Peripheral myelin protein 22 (*Pmp22*), and Myelin‐associated‐glycoprotein (*Mag*).^23^ At the same time, SCs upregulate specific genes in response to nerve injuries, such as the transcription factor *c‐Jun*, an antagonist of *Krox20* expression, and the main driver of the SC‐dependent repair program.^24^ De‐differentiation of SCs by upregulation of *c‐Jun* is a hallmark of repair SCs, a specific cell phenotype essential for in vivo nerve regeneration.^23^ Whether mechanical signals from the substrate stiffness and mechanotransduction modulate the expression of repair SC markers, such as *c‐Jun*, is still unknown. To this end, we investigated the expression levels of *c‐Jun* on SCs in response to different substrate stiffness. We found that levels of nuclear c‐Jun transcription factor are upregulated almost 1.5‐fold in SCs seeded on compliant (1.1 kPa, n = 125) matrices, compared to stiff (27.7 kPa, n = 183) matrices [Figure 3(a–c)]. In addition, the Western blot analysis confirmed the immunocytochemistry results [Figure 3(d)]. Another important regulator that is associated with SC pro‐regenerative capacity is the transcription factor Sox2. The expression of the transcription factor Sox2 has been associated with the maintenance of a pluripotent stem‐cell fate. During PNS development Sox2 is expressed in progenitors and immature SCs, and it is also reexpressed in pro‐regenerative SCs after nerve injury.^25^ We found the levels of Sox2 in nuclei of SCs seeded on stiff matrices (n = 235 cells) to be twice as high compared to those on compliant matrices (n = 200 cells), as determined by immunofluorescence analysis [Figure 3(e–g)]. Increased total protein levels of Sox2 were obtained in Western blots from SCs seeded on stiff matrices [Figure 3(h)]. How the mechanical environment affects SC plasticity and how much it contributes to the direction of differentiated/undifferentiated SC phenotypes during nerve regeneration is not completely understood. In a recent paper, the upregulation of Sox2 expression in cultured RSC 96 SCs is shown to be associated with an increase in the formation of focal adhesions and deposition of fibronectin.^26^ Furthermore, the formation of mature focal adhesions complexes and increase in cell spreading areas are known to scale with matrix rigidity. It remains to be explored, whether stiff substrates promote the formation of focal adhesions and the deposition of fibronectin in SCs, in parallel to their increasing of the SCs spreading areas. On the other hand, c‐Jun and Sox2 are both known to be upregulated after nerve injury. Besides, Sox2 expression is not controlled by c‐Jun, as is the case for its well‐known antagonist expression of the pro‐myelinating transcription factor Krox20.^24^ These results suggest an intricate interplay between matrix stiffness, mechanotransduction, and the modulation of SC stage‐specific markers that requires further investigation. The role of matrix stiffness in the regulation of SC phenotypes is of great interest to the understanding of fundamental aspects of SC physiology and regeneration capacity.

### 2.4 Impact of matrix stiffness on the morphology and outgrowth of adult DRG neurites

**FIGURE 4:**
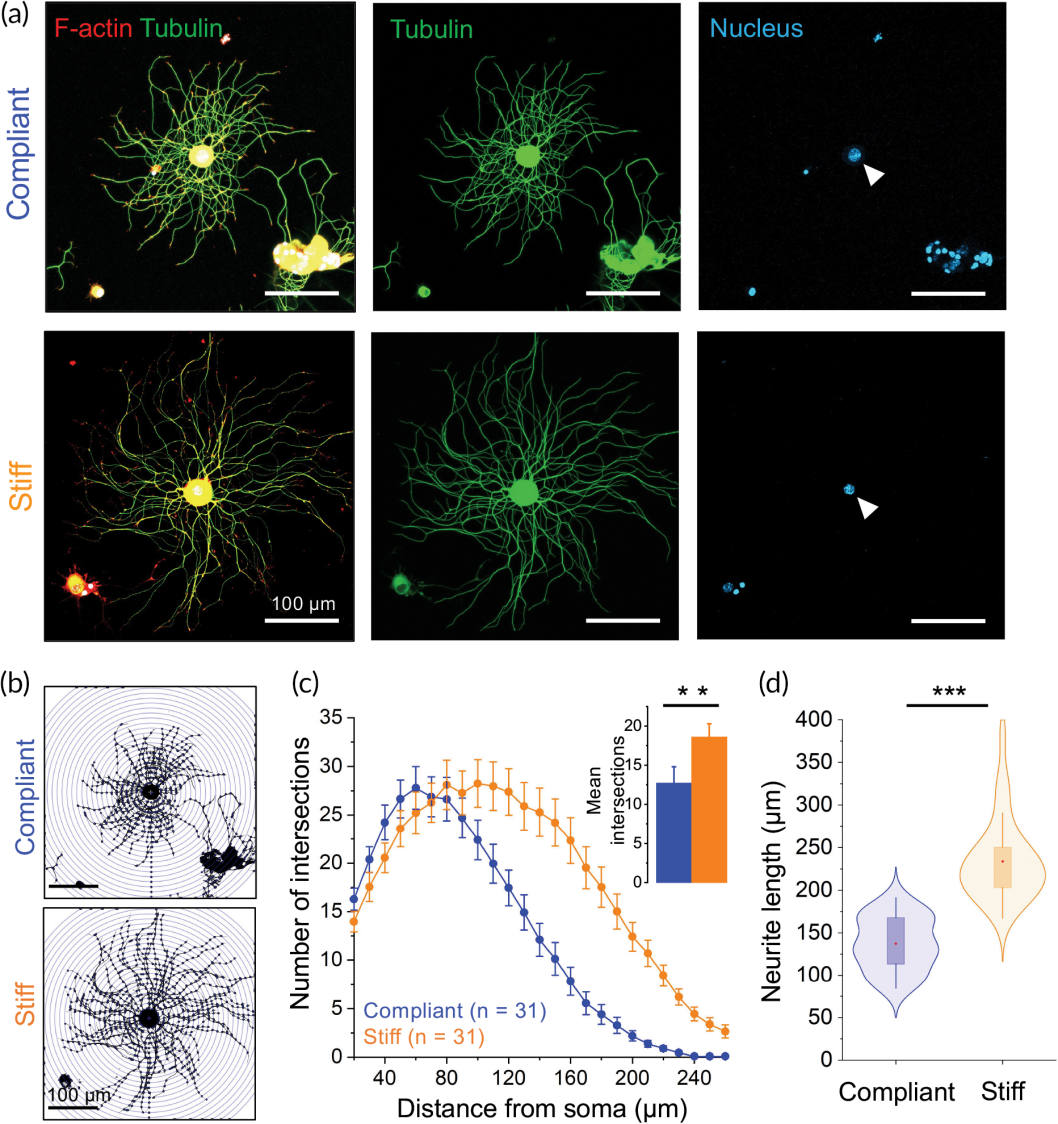
DRG neuron morphology and neurite growth are influenced by substrate stiffness. (a) Representative confocal images showing typical DRG neuron morphologies (green) after 48 hours in culture seeded on compliant (top) and stiff (bottom) laminin‐coated PAAm gel substrates. (b and c) Neuron arborization (Sholl analysis). (d) Quantification of neurite length. Actin cytoskeleton and nucleus labeled in red and blue, respectively. **P < .001 and ***P < .0001, Mann–Whitney test. n = 31 cells for compliant and stiff substrates, respectively. Abbreviation: DRG, dorsal root ganglion

Another crucial aspect of biomedical nerve regeneration using bioengineered scaffolds is the enhancement of neuronal outgrowth. Using 2D elastic substrates of PNS stiffness, we have recently shown in embryonic DRG organotypic explants that neurite elongation is promoted on stiffer substrates compared to compliant substrates.^9^ Embryonic organotypic DRG explants are an excellent, widely accepted in vitro model for the research on PNS development and regeneration. They contain SC progenitors and embryonic fibroblasts in the same preparation, which could influence neuronal outgrowth. Whether physical cues from the local microenvironment directly affect neuronal outgrowth in the absence of other cell types remains elusive. Therefore, we investigated the outgrowth of isolated DRG neurites from adult mice, in response to variation of substrate stiffness analogous to SCs. After 48 hours in vitro, the morphology of single DRG neurons on compliant (1.1 kPa) and stiff (27.7 kPa) laminin‐coated substrates was examined with confocal microscopy [Figure 4(a)]. To characterize the complexity of neuron arborization as a function of substrate stiffness, we implemented the Sholl analysis [Figure 4(b)]. Results from the Sholl analysis show an increased number of neuronal processes in DRG neurons exposed to stiff substrates compared to soft substrates, as indicated by the right shifting of the stiff‐representing curve [Figure 4(c) and inset bar plot]. At closer distances from the neuronal body (between 0 and 60 μm), we observe a similar number of neurites growing from the soma. Finally, the neurite length analysis shows that isolated DRG neurons extend significantly longer neurites (234 ± 8 μm, n = 31) when exposed to substrates of increased stiffness compared to compliant substrates (137 ± 6, n = 31) [Figure 4(d)].

## 3 CONCLUSIONS

The phenotype of SCs and the expression of key transcription regulatory factors that drive development and myelination among others are sensitive to changes in the stiffness of the ECM. Likewise, the intracellular distribution of transcription regulatory factors changes in response to alterations in the ECM stiffness. The observations made in the present study refine our understanding of the PNS physiology and pathophysiology and may be exploited in biomedical research areas. The intrinsic physiological plasticity of SCs, which change their phenotype in response to physiological and pathophysiological changes of their microenvironment, in conjunction with their demonstrated mechanosensitivity render them powerful targets for cell‐based regenerative therapies. For example, SCs may be transplanted into bioengineered scaffolds for PNS or CNS repair, whose biomechanical properties may be appropriately adjusted to drive SCs to the desired phenotype, and eventually promote nervous system regeneration.

## 4 MATERIALS AND METHODS

All chemicals were obtained from Sigma‐Aldrich unless otherwise stated.

### 4.1 Preparation of PAAm substrates

PAAm gels within the physiological nerve stiffness range were produced as previously described protocols.^9^, ^27^ Compliant (1.1 kPa) and stiff (27.7 kPa) gels with a thickness of 150 μm were incubated with poly‐d‐lysine overnight at 4°C followed by 2 hours incubation with 10 μg/mL laminin. Substrates were covered with cell culture medium and allowed to equilibrate at 37°C for 1 hour before seeding of cells. The laminin coating homogeneity on the PAA substrates and the consistency of coating between the two stiffnesses were tested by immunochemistry and confocal microscopy (see supporting information Figure S1).

### 4.2 SC isolation, purification, and maintenance

Experiments were performed in accordance with animal welfare laws, complied with ethical guidelines and were approved by the responsible local committees and government bodies (University of Erlangen, Amt für Veterinärwesen der Stadt Erlangen and the Regierung von Unterfranken, TS‐00/12). SCs were isolated from Wistar adult rats as previously described.^9^ Cells were expanded in proliferating medium: DMEM, 10% FBS (HyClone), 10 ng/mL Neuregulin (Peprotech), 2 mM Glutamax (Invitrogen), 2 μM Forskolin, and incubated at 37°C in 5% CO_2_. After expansion, SCs were seeded on laminin‐coated PAAm gels containing DMEM, 10% FBS (HyClone), 10 ng/mL Neuregulin, 2 mM Glutamax, and incubated for 48 hours at 37°C in 5% CO_2_. SCs in experiments were not used beyond passage 5.

### 4.3 DRG culture

Adult (4–6 months old) C57BL/6J mice were killed by cervical dislocation and spinal cords were removed. DRGs were dissected and incubated in Neurobasal medium (NB, Invitrogen) containing 2.5 mg/mL collagenase and incubated for 1 hour at 37°C in 5% CO_2_. Then, the tissue was homogenized using fired‐polished glass pipettes and DRG neurons were separated from axon stumps and myelin debris by generating a 14% bovine serum albumin layer and centrifuged for 8 minutes at 120 rpm. The pellet with DRG neurons was resuspended in NB containing 20 μL/mL B27 supplement 50× (Gibco), 2 mM Glutamax, 10 μm/mL antibiotic‐antimycotic, 0.01 μg/mL nerve growth factor (Invitrogen) and seeded on the PAAm substrates. DRG neurons were kept in an incubator at 37°C in 5% CO_2_.

### 4.4 Immunofluorescence and image analysis

Either SCs or DRG neurons were seeded on laminin‐coated PAAm substrates and maintained for 48 hours before fixation for 20 minutes with 4% PFA and processed for immunocytochemistry. Cells were incubated with primary antibodies (anti‐YAP 1:250, anti‐Krox20 1:500, anti‐Oct6, anti‐c‐Jun 1:200, anti‐Sox2 1:500, anti‐beta‐tubulin 1:1000) overnight at 4°C. Secondary antibodies (1:1000), rhodamine‐phalloidin (1:150), and DAPI were incubated for 2 hours at 37°C. A reference list of used antibodies is summarized in supporting information, Table 1. Fluorescent images of SCs were acquired at 40× using an LSM 980 Zeiss (Carl Zeiss, Germany) confocal microscope. Images of 0.5 μm spacing z‐stacks from random areas were obtained and imported to FIJI (NIH, USA). For quantitative fluorescent intensity analysis of transcription factors Krox20, Oct6, c‐Jun, and Sox2 inside the SC nuclei, the nuclei were identified using the DAPI channel and the “region of interest” (ROI) tool. The ROIs for the nuclei were added to the ROI manager, and then the fluorescent intensity for Krox20, Oct6, c‐Jun, and Sox2 was measured on the corresponding Alexa‐488 channel within the nuclear regions. Background fluorescence was subtracted from intensity values. The quantification of nucleus/cytoplasmic YAP ratio was calculated using the following equation:

$$\mathrm{YAP}\;\mathrm{nuc}/\text{cyto ratio}=\frac{\sum_{\mathrm{nuc}}^{\mathrm{int}}/A.\mathrm{nuc}}{\sum_{\mathrm{nuc}}^{\mathrm{int}}/A.\text{cyto}}$$

where $\sum_{\mathrm{nuc}}^{\mathrm{int}}$ and $\sum_{\text{cyto}}^{\mathrm{int}}$ represent the sum of the intensity values for the pixels in the nucleus and the cytoplasmic regions, respectively, and *A. nuc* and *A. cyto* represent the area of the corresponding regions. Subtract background tool was applied before fluorescent intensity analysis.

### 4.5 Neurite length and Sholl analysis

The NeuronJ plugin on FIJI was implemented for neurite tracing. Tracings were measured from the growth cones to the cell soma for each neuron. For the Sholl analysis, fluorescent images were converted to binary, and neurite arbor complexity was quantified in nonoverlapping neurons utilizing the Sholl Analysis plugin (FIJI). A starting radius of 20 μm and an outer radius of 260 μm were selected to cover the distance of the longest neurite. The distance between consecutive radiuses (radius step size) was set to 10 μm. The number of crossing neurites to each circle was quantified for each neuron and the data exported for analysis.

### 4.6 Western blot

Samples were prepared by lysing the SCs in RIPA buffer (Thermo Fisher) containing protease inhibitors. Proteins were separated on a 4%–20% SDS‐PAGE (BIO‐RAD). Western blotting was performed using primary antibodies anti‐Krox20 (1:2000), anti‐Oct6 (1:2000), anti‐c‐Jun (1:2000), and anti‐Sox2 (1:2500). An antibody against actin (1:2000) was used as a loading control. Secondary peroxidase‐conjugated (HRP) antibodies were diluted 1:2000 in 3% fat‐reduced milk. Images were acquired using G:Box Chemi XX9 system (Syngene, UK). See Table 2 in supporting information for analysis of protein bands.

### 4.7 Statistical analysis

Data were exported to Origin Pro 9 software. The results are considered statistically significant when *P*‐value <.05. *P* values in figures are represented by (*) *P* < .05, (**) *P* < .001, and (***) *P* < .0001. Data are presented as mean values ± SEM.

## CONFLICT OF INTEREST

The authors declare no conflict of interest.

## AUTHOR CONTRIBUTIONS

**Gonzalo Rosso:** Conceptualization (lead); formal analysis (lead); investigation (lead); methodology (lead); project administration (equal); writing – original draft (lead); writing – review and editing (lead). **Daniel Wehner:** Formal analysis (supporting); investigation (supporting); writing – original draft (supporting). **Christine Schweitzer:** Investigation (supporting). **Stephanie Möllmert:** Formal analysis (supporting). **Elisabeth Sock:** Investigation (supporting); resources (supporting); writing – original draft (supporting). **Jochen Guck:** Conceptualization (supporting); resources (supporting); supervision (supporting); writing – original draft (supporting).

## Supporting information


**Appendix** **S1**: Supporting Information.
