# Supplementary material for: Matrix stiffness mechanosensing modulates the expression and distribution of transcription factors in Schwann cells
Source: Bioeng Transl Med. 2021 Sep 21;7(1):e10257. doi: 10.1002/btm2.10257 (PMC8780053; doi:10.1002/btm2.10257)
Supplement: Supplementary file 1 — Appendix S1: Supporting Information. [file BTM2-7-e10257-s001.doc]

**Supporting information**

**Matrix stiffness mechanosensing modulates the expression and distribution of transcription factors in Schwann cells.**

*Gonzalo Rosso, Daniel Wehner, Stephanie Möllmert, Christine Schweitzer, Elisabeth Sock, Jochen Guck and Victor Shahin*

*Polyacrylamide gel functionalization and fluorescent image analysis*

Laminin-coated PAAm gels were incubated overnight with an antibody against laminin (L9393, Sigma-Aldrich) followed by secondary Alexa-488 secondary antibody incubation. Maximum intensity projection images were generated by imaging 10 μm thick confocal planes of 0.5 μm z-stacks from below and above the gel surface. Three different randomly selected areas from each independent gel were imaged and fluorescent intensities were measured with FIJI (NIH) and plotted in histograms to compare the distribution of the laminin coating (Supplementary figure 1a).

*Polyacrylamide gel elasticity measurements*

The elasticity of PAAm gels was measured using an AFM system (JPK, Nanowizard 4). A 10 μm in diameter polystyrene bead was glued at the end of an Arrow-TL1 tip-less cantilever (NanoWorld). The cantilever spring constant was measured using the thermal noise method and values were ranged between 0.02 - 0.05 N/m. The average values of PAAm gels of different stiffness were obtained from 4 different randomly selected regions across the gels surface (Supplementary figure 1b). For each region, a total of 9 force-spectroscopy curves were taken inside a 60 μm x 60 μm square grid. Force curves were analyzed using the JPK manufacturer's software by applying the Hertz model.


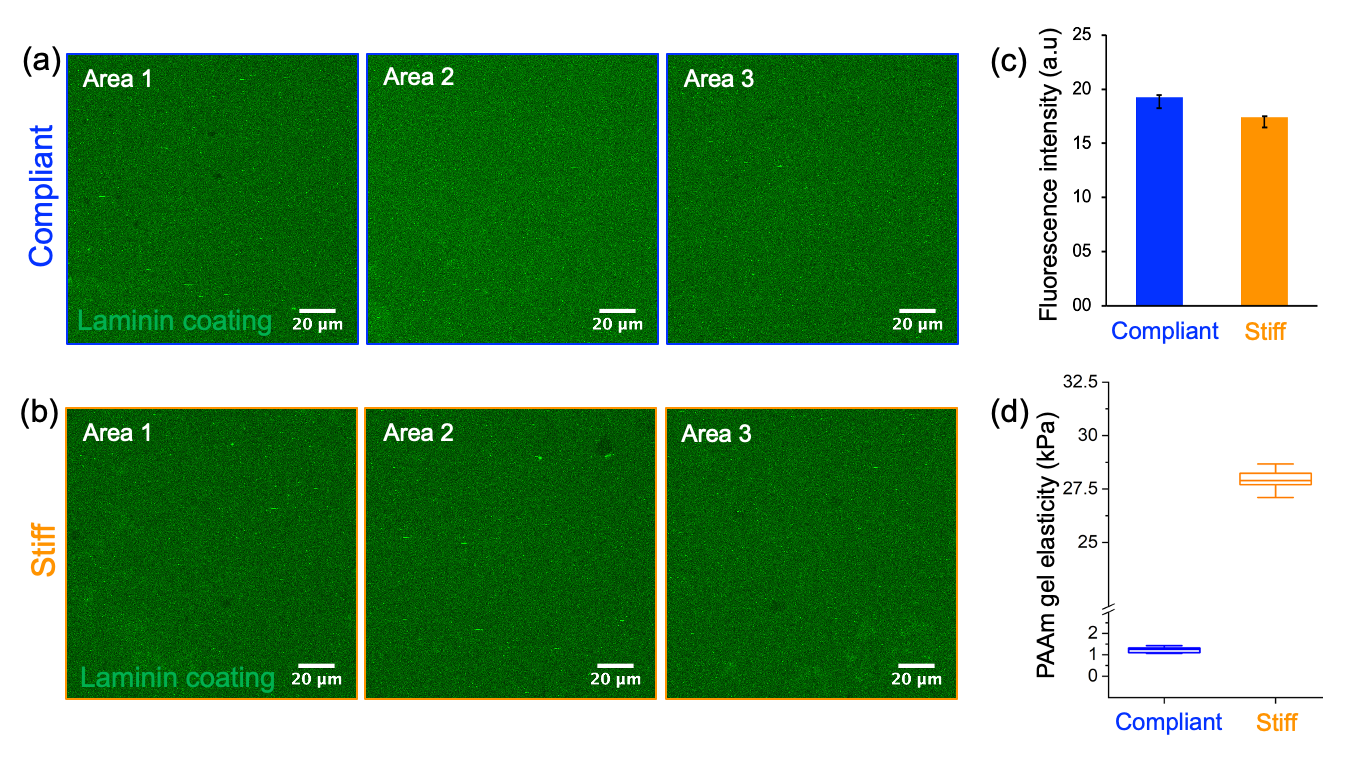


Supplementary Figure 1. Laminin functionalization of PAAm gels and mechanical characterization. (a and b) Representative confocal images showing functionalized PAAm gels with 10 ug/ml of laminin and visualized through immunohistochemistry using an anti-laminin antibody (1:50). (c) Quantification of PAAm substrate functionalization with laminin by immunofluorescence. (d) Graph showing PAAm bulk substrate elasticity measured on compliant (1.1 ± 0.1 kPa) and stiff (27.7 ± 0.3 kPa) gels using AFM.

Table 1. *List of antibodies and fluorescent probe used for immunostainings and Western blot*

| **Antibody** | **Company/source** | **Catalogue #/Reference** |
| --- | --- | --- |
| Anti-YAP | Santa Cruz | sc-101199 |
| Anti-Krox20 | * | Ref. 1. Fröb et al., 2019 |
| Anti-Oct6 | Santa Cruz | sc-376143 |
| Anti-c-Jun | Invitrogen | 39-7500 |
| Anti-Sox2 | * | Ref. 2. Thein et al., 2010 |
| Anti-beta-tubulin | Covance | MMS-435P |
| Anti-laminin | Sigma-Aldrich | L9393 |
| Anti-beta actin | Abcam | ab8226 |
| Goat anti-Rabbit IgG (HRP) | Abcam | ab97080 |
| Goat anti-Mouse IgG (HRP) | Abcam | ab205719 |
| Goat anti-Mouse IgG Alexa 488 | ThermoFisher | A-10680 |
| Goat anti-Rabbit Alexa 488 | ThermoFisher | A-11008 |
| Rhodamine-phalloidin | ThermoFisher | R415 |

(*) These homemade antibodies were produced in the laboratory of Prof. Dr. Michael Wegner (FAU Erlangen-Nuremberg).

Ref.1. Guinea pig anti-Krox20 antiserum (generated against a bacterially expressed and purified peptide corresponding to amino acids 28–166 of mouse Krox20 according to accession number NM_010118.3. Citation: Fröb, F., Sock, E., Tamm, E.R. et al. Ep400 deficiency in Schwann cells causes persistent expression of early developmental regulators and peripheral neuropathy. Nat Commun 10, 2361 (2019). https://doi.org/10.1038/s41467-019-10287-w

Ref 2. Rabbit anti-Sox2 antiserum (generated against a bacterially expressed and purified peptide corresponding to amino acids 10–38 of mouse Sox2 according toaccession numberNM_011443. Citation: Thein DC, Thalhammer JM, Hartwig AC, et al. The closely related transcription factors Sox4 and Sox11 function as survival factors during spinal cord development. Journal of Neurochemistry. 2010 Oct;115(1):131-141. DOI: 10.1111/j.1471-4159.2010.06910.x.

*Western blot statistical analysis*

The levels of Krox20, Oct6, Sox2 and c-Jun proteins in Western blots were analyzed with FIJI (NIH). Data in Table 2 are presented as mean gray values ± standard error of the mean (SEM). Significant statistical differences are indicated by (*) (p <0.05, one-way analysis of variance followed by Bonferroni’s multiple comparison test) between compliant and stiff substrates. The number of blots for each protein (N) is greater or equal to 3.

Table 2.

|  | **Krox20** |  |
| --- | --- | --- |
| **Compliant** | 0.52 ± 0.2 | n.s |
| **Stiff** | 0.7 ± 0.3 |
|  |  |  |
|  | **Oct6** |  |
| **Compliant** | 0.75 ± 0.1 | * |
| **Stiff** | 1.26 ± 0.2 |
|  |  |  |
|  | **Sox2** |  |
| **Compliant** | 1.20 ± 0.2 | * |
| **Stiff** | 1.72 ± 0.1 |
|  |  |  |
|  | **c-Jun** |  |
| **Compliant** | 1.85 ± 0.1 | * |
| **Stiff** | 1.28 ± 0.2 |
